# Sequential broncho-alveolar lavages reflect distinct pulmonary compartments: clinical and research implications in lung transplantation

**DOI:** 10.1186/s12931-018-0786-z

**Published:** 2018-05-25

**Authors:** Liran Levy, Stephen C. Juvet, Kristen Boonstra, Lianne G. Singer, Sassan Azad, Betty Joe, Marcelo Cypel, Shaf Keshavjee, Tereza Martinu

**Affiliations:** 1 Toronto Lung Transplant Program, University Health Network, University of Toronto, Toronto, ON Canada; 2 Toronto General Hospital, 585 University Ave, PMB 11-128, Toronto, ON M5G 2N2 Canada

**Keywords:** Bronchoalveolar lavage, Lung transplant, Cells, Proteins, Cytokines

## Abstract

**Background:**

Bronchoalveolar lavage (BAL) has proven to be very useful to monitor the lung allograft after transplantation. In addition to allowing detection of infections, multiple BAL analytes have been proposed as potential biomarkers of lung allograft rejection or dysfunction. However, BAL collection is not well standardized and differences in BAL collection represent an important source of variation. We hypothesized that there are systematic differences between sequential BALs that are relevant to BAL analysis.

**Methods:**

As part of 126 consecutive bronchoscopies in lung transplant recipients, two sequential BALs (BAL1 and BAL2) were performed in one location during each bronchoscopy by instilling and suctioning 50 ml of normal saline twice into separate containers. Cell concentration, viability and differentials, Surfactant Protein-D (SP-D), Club Cell Secretory Protein (CCSP), and levels of CXCL10, IL-10, CCL2, CCL5, VEGF-C, RAGE, CXCL9, CXCL1, IL-17A, IL-21, PDGF, and GCSF were compared between BAL1 and BAL2.

**Results:**

Total cell concentration did not differ between BAL1 and BAL2; however, compared to BAL2, BAL1 had more dead cells, epithelial cells, neutrophils, and higher concentrations of airway epithelium-derived CCSP and inflammatory markers. BAL2 had a higher concentration of SP-D compared to BAL1.

**Conclusion:**

In this study performed in lung transplant recipients, we show that sequential BALs represent different lung compartments and have distinct compositions. BAL1 represents the airway compartment with more epithelial cells, neutrophils, and epithelium-derived CCSP. Conversely, BAL2 samples preferentially the distal bronchoalveolar space with greater cell viability and higher SP-D. Our findings illustrate how the method of BAL collection can influence analyte concentrations and further emphasize the need for a standardized approach in translational research involving BAL samples.

**Electronic supplementary material:**

The online version of this article (10.1186/s12931-018-0786-z) contains supplementary material, which is available to authorized users.

## Background

Bronchoalveolar lavage (BAL) is a technique widely used in pulmonary medicine and lung transplantation to diagnose lung infections and other processes or evaluate treatment effects [1, 2]. Examination of the cellular composition and protein constituents in the BAL provides a unique window into the microenvironment of the lung. In lung transplantation, BAL proteins have been proposed as potential biomarkers of acute rejection [3–6] and chronic lung allograft dysfunction (CLAD) [7]. However, small sample sizes, lack of control for potential confounders and lack of standardization related to BAL collection and handling have all been proposed as sources of variability between studies [7, 8].

Although BAL has been used as a research tool in lung transplantation for decades, the technique varies markedly between centers. In an informal survey conducted by our group among 25 lung transplant centers from 14 countries, BAL collection ranged from 1 to 6 sequential lavages of 20-100 ml each with inconsistent pooling prior to analysis (Additional file 1: Table S1). In an attempt to create a common approach to BAL collection, BAL standardization guidelines were published by the European Respiratory Society in 1999 [2], and guidelines specific to patients with interstitial lung diseases were put forth by the American Thoracic Society in 2012 [9]. While these documents set an important precedent, they leave room for significant variability in BAL collection and processing. Neither the optimal total volume nor the number of aliquots to be instilled has been established.

There are specific issues related to performing BAL in lung transplant patients. Poor long-term outcomes after lung transplantation create a strong mandate for research, patient enrollment, and multi-center collaboration. Standardization of BAL collection and processing is essential to enable sharing of data across collaborative research networks and to maximize their utility. The purpose of this study was to determine whether there are systematic differences between sequential BAL fractions in lung transplant patients, with a focus on cellular composition and proteins that have previously been shown to correlate with clinical outcomes in lung transplant recipients, including lymphocytes [10], neutrophils [7, 10], Surfactant Protein-D (SP-D) [11], Club Cell Secretory Protein (CCSP) [12, 13], CXCL10 [7], IL-10 [14], CCL2 [7], CCL5 [7], VEGF-C [15], RAGE [16], CXCL9 [7], CXCL1 [17], IL-17A [18], IL-21 [19], PDGF [20–22] and GCSF [23].

## Methods

### Patient selection and sample collection

This is a retrospective single-center cohort study based on prospectively collected BAL samples and clinical information, approved by the Institutional Research Ethics Board. The study population consisted of all consented lung transplant recipients at Toronto General Hospital who underwent a bronchoscopy between August and October 2015. Immunosuppression, antimicrobial prophylaxis, and treatment of acute rejection were administered for all patients in accordance with the Toronto Lung Transplant Program protocol as described previously [24, 25]. CLAD was defined as a sustained (at least 3 weeks) and irreversible decline in FEV1 to ≤80% of the post-transplant baseline, which was itself defined as the average of the two highest FEV1 values at least 3 weeks apart, in the absence of other etiologies [26]. BAL samples from 126 consecutive bronchoscopies were collected and analyzed as detailed below.

### Bronchoscopy procedure and BAL collection

**Fig. 1:**
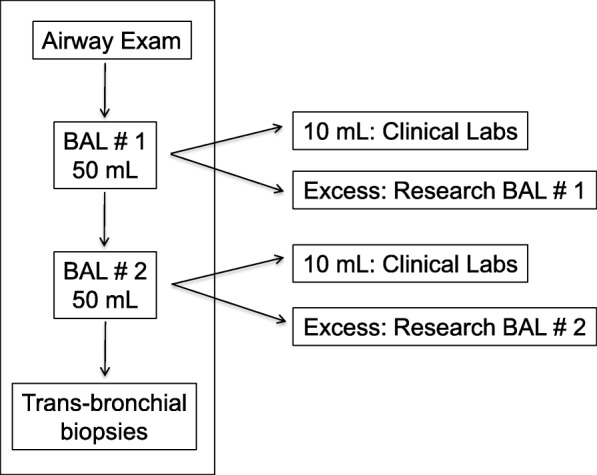
Standardized bronchoalveolar lavage collection protocol and measurement of return volumes in lung transplant recipients. After initial airway inspection, a bronchoalveolar lavage (BAL) is performed by wedging the bronchoscope in a lung segment, instilling 50 mL of saline followed by aspiration (BAL1), and repeating the instillation of another 50 mL of saline followed by aspiration (BAL2). BAL1 and BAL2 samples are collected into separate containers. 10 mL of each BAL1 and BAL2 were sent to the clinical laboratory. The remaining volume of each of BAL1 and BAL2 was transferred to the research laboratory for further analysis

At our center, scheduled surveillance bronchoscopies are carried out at 0.5, 1.5, 3, 6, 9, 12, 18 and 24 months post-transplant. Additional diagnostic bronchoscopies are performed if clinically indicated. Bronchoscopies were conducted via the oropharyngeal route under conscious sedation. Supplemental oxygen was provided to maintain an oxygen saturation of > 90% (intubation was not routinely performed). Pharyngeal anesthesia with 4% lidocaine was applied to the upper airways prior to the bronchoscopy, and intravenous Midazolam and Fentanyl were administered prior to the bronchoscopy for sedation. In addition, 1% lidocaine was administered to the trachea and mainstem airways during the bronchoscopy for local anesthesia of the airway mucosa. Blood pressure, heart rate, oxygen saturation, electrocardiogram and consciousness level of the patient were continuously monitored. After passing through the upper airways, avoiding suctioning as much as possible so as not to contaminate the bronchoscope, an initial airway inspection was carried out. As part of our institutional protocol aimed to measure markers of aspiration in the airways, a large airway bronchial wash (LABW) was performed in the mainstem bronchus with instillation and subsequent suctioning of 20 ml of normal saline (this sample was not assessed in this study). The bronchoscope was then placed in a wedged position within the targeted segment; Per protocol, when no particular location was targeted, BAL sampling was conducted in the right middle lobe or left upper lobe (preferentially the lingula) of the lung allograft, as suggested by the ATS/ERS guidelines [9]. In the case of localized disease processes, the targeted segment was chosen based on radiological examination or visual inspection. After achieving a wedged position with the bronchoscope, 50 ml of normal saline were instilled and then suctioned while maintaining the wedged position (BAL1). This procedure was immediately repeated once again (BAL2). BAL1 and BAL2 samples were collected into separate containers, and the return volumes were recorded. From BAL1, 10 ml was sent for general clinical microbiologic analysis. From BAL2, 10 ml was sent for clinical cytology analysis. All remaining fluid was processed and stored for research. Transbronchial biopsies (if any) were performed after BALs. Suctioning throughout the procedure was performed using a wall-mounted suction system (see protocol in Fig. 1).

### Processing of BAL samples

After separation of clinical samples, as described above, BAL1 and BAL2 were placed on ice and processed within three hours of sample collection. Cell concentrations, differentials, and cell viability were measured as described below, in aliquots of fresh whole (raw) BAL samples that were separated prior to further processing. Subsequently, BAL1 and BAL2 samples were centrifuged for 20 min at 3184G at 4 °C. The supernatant was carefully transferred into separate tubes and stored at − 80 °C. The cell pellets were stored as part of our ongoing biobanking and not used in this study.

### Assessment of cell counts

Cell concentration for all 126 BAL1-BAL2 sample pairs were assessed using an automatic Vi-Cell XR Cell Viability Analyzer (Beckman Coulter, Mississauga, ON, Canada). For confirmation, a manual cell count using trypan blue dead cell exclusion on a hemocytometer was performed on nine randomly selected sample pairs with quantification of ciliated epithelial cells. Cytospin preparations of 6 of these sample pairs were made: 150uL of the whole (raw) BAL was loaded into a Cytospin filter attached to a charged glass slide. The Cytospin filters with glass slides were then centrifuged at 800 rpm for 3 min (Shandon Cytospin 4 Centrifuge, ThermoFisher). The slides were then air dried and stained with HARLECO® Hemacolor® (EMD Chemicals, NJ, USA). Percentages of polymorphonuclear granulocytes, eosinophils, macrophages, and lymphocytes were quantified. All manual sample analyses were performed by experienced operators blinded to sample group.

### BAL protein analysis

The first 20 paired BALs of the cohort were arbitrarily chosen for analysis of CCSP and SP-D. Stored frozen BAL supernatants were thawed on ice. CCSP and SP-D were measured by enzyme-linked immunosorbent assay (ELISA) (DuoSet® ELISA; R&D Systems, Minneapolis, MN, USA). All samples and standards were run in duplicate according to manufacturers’ protocols. The 15 pairs of BAL that were collected sequentially after the first 20 (and were therefore not previously thawed) were used to assess inflammatory markers: A custom multiplex bead kit (R&D Systems) was used to measure CXCL10, IL-10, CCL2, CCL5, VEGF-C, RAGE, CXCL9, CXCL1, IL-17A, IL-21, PDGF and GCSF based on manufacturer’s instructions. Samples and standards were run in duplicate. Biomarker concentrations were obtained using a Bio-Plex® MAGPIX™ Multiplex reader (Bio-Rad Laboratories, Hercules, CA). For all analytes, any value falling below the lower limit of detection was assigned a level of 0 ng/ml.

### Statistical analyses

Comparisons of the paired BAL samples were performed using a non-parametric Wilcoxon matched-pairs signed rank test. Results are shown as median [interquartile range 25–75%]. Spearman correlation was used to evaluate the pairing of BAL1 and BAL2, yielding a Spearman’s correlation coefficient (*r*). All statistical analyses were performed with GraphPad Prism version 5.01 software (GraphPad Software, La Jolla, CA, USA). A *p*-value < 0.05 was considered statistically significant.

## Results

### Characteristics of patients and bronchoscopies

One hundred twenty-two patients underwent 126 sequential bronchoscopies. Baseline characteristics are detailed in Table 1 and clinical characteristics at the time of the bronchoscopies are outlined in Table 2. Data for the overall cohort as well as for each sub-analysis cohort is shown separately. The majority of the BAL samples were obtained during surveillance bronchoscopies and were obtained from the RML. These proportions were similar between the different sub-cohorts. Less than a quarter of the BALs were infected.

**Table 1:** Baseline patient characteristics

		Sub-cohorts used for:		
	Main cohort *N* = 122^a^	CCSP & SP-D *N* = 19^a^	Inflammatory proteins *N* = 15	Cell count *N* = 6
Recipient age at transplant, year (Mean ± SD)	54.1 ± 16.7	55.3 ± 15.8	57.7 ± 11	56.7 ± 16.7
Primary diagnosis				
Idiopathic pulmonary fibrosis	53 (43.4%)	10 (52.6%)	7 (46.7%)	5(83.3%)
Chronic obstructive pulmonary disease	23 (18.9%)	3 (15.8%)	4 (26.7%)	0
Cystic fibrosis	16 (13.1%)	3 (15.8%)	3 (20%)	0
Scleroderma	6 (4.9%)	1 (5.3%)	0	0
Chronic Lung Allograft Dysfunction	5 (4.1%)	0	0	0
Sarcoidosis	4 (3.3%)	0	0	0
Bronchiectasis	4 (3.3%)	0	1 (6.7%)	0
Alpha-1 antitrypsin deficiency	3 (2.5%)	0	0	0
Langerhans cell histiocytosis	2 (1.6%)	1 (5.3%)	0	0
Other	6 (4.9%)	1 (5.3%)	0	1 (16.7%)
Transplant type				
Single lung transplantation	23 (18.9%)	5 (26.3%)	5 (33.3%)	1 (16.7%)
Bilateral lung transplantation	99 (81.1%)	14 (73.7%)	10 (66.7%)	5 (83.3%)
Male Gender	69 (56.6%)	14 (73.7%)	9 (60%)	3 (50%)
Number of lung transplants				
First lung transplant	116 (95.1%)	18 (94.7%)	15 (100%)	5 (83.3%)
Second lung transplant	6 (4.9%)	1 (5.3%)	0	1 (16.7%)
CLAD status at time of bronchoscopy				
CLAD	14 (11.5%)	7 (36.8%)	3 (20%)	1 (16.7%)
No CLAD	108 (88.5%)	12 (63.2%)	12 (80%)	5 (83.3%)

AR=Acute rejection; n=number; %=percent of samples

^a^Number of patients is smaller than number of bronchoscopies

**Table 2:** Bronchoscopy characteristics

		Sub-cohorts used for:		
	Main cohort *N* = 126*	CCSP & SP-D *N* = 20*	Inflammatory proteins *N* = 15*	Cell count *N* = 6*
Median time from transplant to bronchoscopy, days, Median (IQR)	279 (99.5–553)	189 (95–316)	196 (67.5–285)	189 (93.8–280.5)
Indication for bronchoscopy				
Surveillance	104 (82.5%)	15 (75%)	11 (73.3%)	5 (83.3%)
Diagnostic	22 (17.5%)	5 (25%)	4 (26.7%)	1 (16.7%)
BAL localization				
RUL	5 (4%)	2 (10%)	1 (6.7%)	0
RML	103 (81.7%)	15 (75%)	11 (73.3%)	4 (66.7%)
RLL	4 (3.2%)	1 (5%)	1 (6.7%)	1 (16.7%)
LUL	8 (6.3%)	2 (10%)	1 (6.7%)	1 (16.7%)
LLL	2 (1.6%)	0	1 (6.7%)	0
Missing records	4 (3.2%)	0	0	0
FEV_1_ at the time of bronchoscopy, L (Median, IQR)	2.2 (1.6–2.8)	2 (1.7–3.1)	2 (1.8–2.25)	2 (1.8–2.4)
Presence of clinically-relevant pathogen, number	29 (23%)	6 (30%)	2 (13.3%)	1 (16.7%)
AR grade				
AX	15 (11.9%)	4 (20%)	1 (6.7%)	2 (33.3%)
A0	76 (60.3%)	13 (65%)	9 (60%)	4 (66.7%)
A1	22 (17.5%)	3 (15%)	4 (26.7%)	0
≥ A2	0	0	0	0
Biopsy not done	13 (10.3%)	0	1 (6.7%)	0

AR=Acute rejection; N=number; %=percent of samples

*N represents the number of bronchoscopies

### The volume of recovered BAL fluid was higher for BAL2 than for BAL 1

**Fig. 2:**
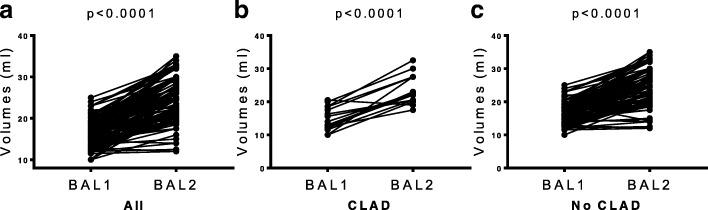
Bronchoalveolar lavage (BAL) return volumes in lung transplant recipients. Volume return after a second 50 ml instillation was significantly higher than following the first 50 ml instillation (15.5 ml (13.4, 18.4) vs. 25 ml (21.0, 30.0), for BAL1 and BAL2 respectively, *p* < 0.0001) **a**. This finding remained consistent when comparing BAL1 and BAL2 in patients with CLAD (14 mL (12.0, 18.5) vs. 22.5 mL (20.5, 27.5) for BAL1 and BAL2 respectively, *p* < 0.0001) **b** or No CLAD (15.6 mL (13.96, 18.35) vs. 25 mL (20, 30) for BAL1 and BAL2 respectively, *p* < 0.0001) **c**. A non-parametric Wilcoxon matched-pairs signed rank test was used for the comparison

Fluid aspirated after the second 50 ml instillation was significantly higher compared to the first instillation (15.5 ml (13.4, 18.4) vs. 25 ml (21.0, 30.0), for BAL1 and BAL2 respectively, *p* < 0.0001) (Fig. 2). In light of the potential impact of CLAD status on volume recovery, we investigated differences between BAL1 and BAL2 separately among CLAD and No CLAD patients. Once again, BAL2 demonstrated higher return volume compared to BAL1 in both group subsets (CLAD: 14 ml (12.0, 18.5) vs. 22.5 ml (20.5, 27.5) for BAL1 and BAL2 respectively, *p* < 0.0001. No CLAD: 15.6 ml (13.96, 18.35) vs. 25 ml (20, 30) for BAL1 and BAL2 respectively, *p* < 0.0001) (Fig. 2).

### Overall cell concentration does not significantly differ between BAL1 and BAL2, but viability is higher in BAL2

**Fig. 3:**
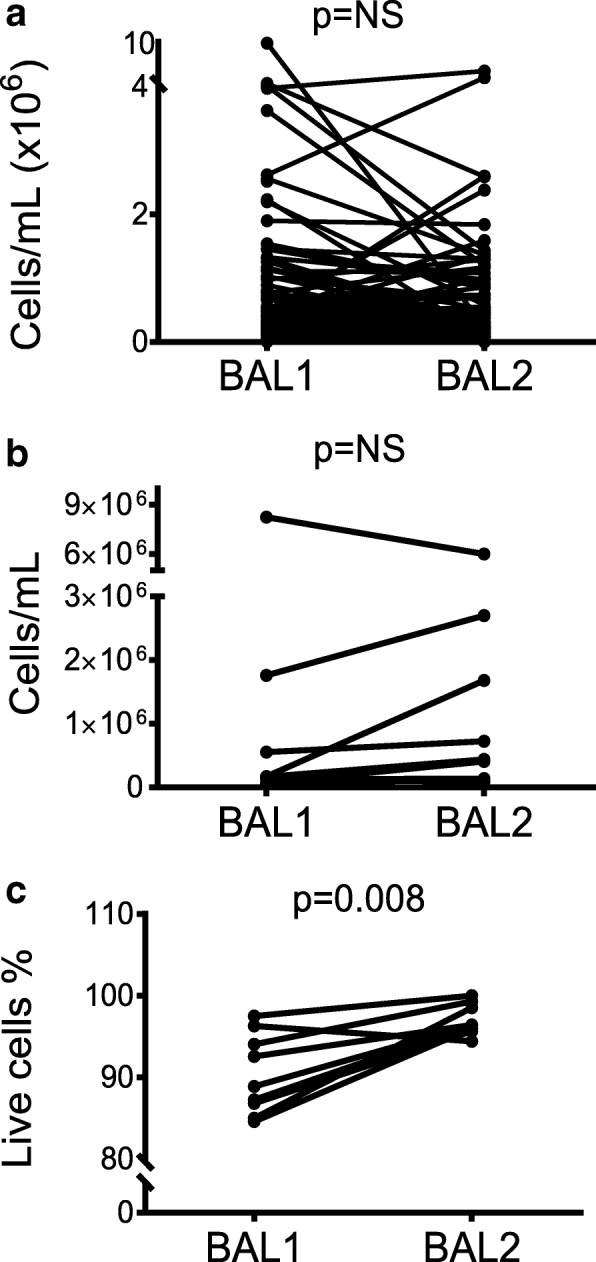
Cell Concentration and cell viability in sequential bronchoalveolar lavages performed in lung transplant recipients. Two sequential bronchoalveolar lavages (BALs) were performed in consecutive lung transplant recipients. Cell concentration was measured using an automated cell counter. Cell concentrations did not differ between BAL1 and BAL2 (*p* = 0.09), which was also confirmed by manual cell count in nine paired BALs (*p* = 0.2) (**a**), (**b**), however cell viability was significantly higher in BAL2 (*p* < 0.05) (**c**). A non-parametric Wilcoxon matched-pairs signed rank test was used for comparison

Cell concentration and differential counts of BAL1 and BAL2 are listed in Additional file 1: Table S2. Overall, BAL cellularity, expressed as cells per milliliter of BAL, did not differ between BAL1 and BAL2 (0.32 × 10^6^/ml (0.17, 0.67) vs. 0.27 × 10^6^/ml (0.16, 0.54), for BAL1 and BAL2 respectively, *p* = 0.09) (Fig. 3a). Cell concentration was confirmed and viability was assessed by manual cell counts on nine paired BALs (Fig. 3b and c). Cell viability, expressed as percent of live cells out of total cells, was significantly higher in BAL2 compared to BAL 1 (88.89% (85.92, 95.18) vs. 96.43% (95.83, 98.92), for BAL1 and BAL2 respectively, *p* < 0.05) (Fig. 3c).

### BAL1 is characterized by higher counts of neutrophils and airway epithelial cells, but lower proportion of alveolar macrophages compared with BAL2

**Fig. 4:**
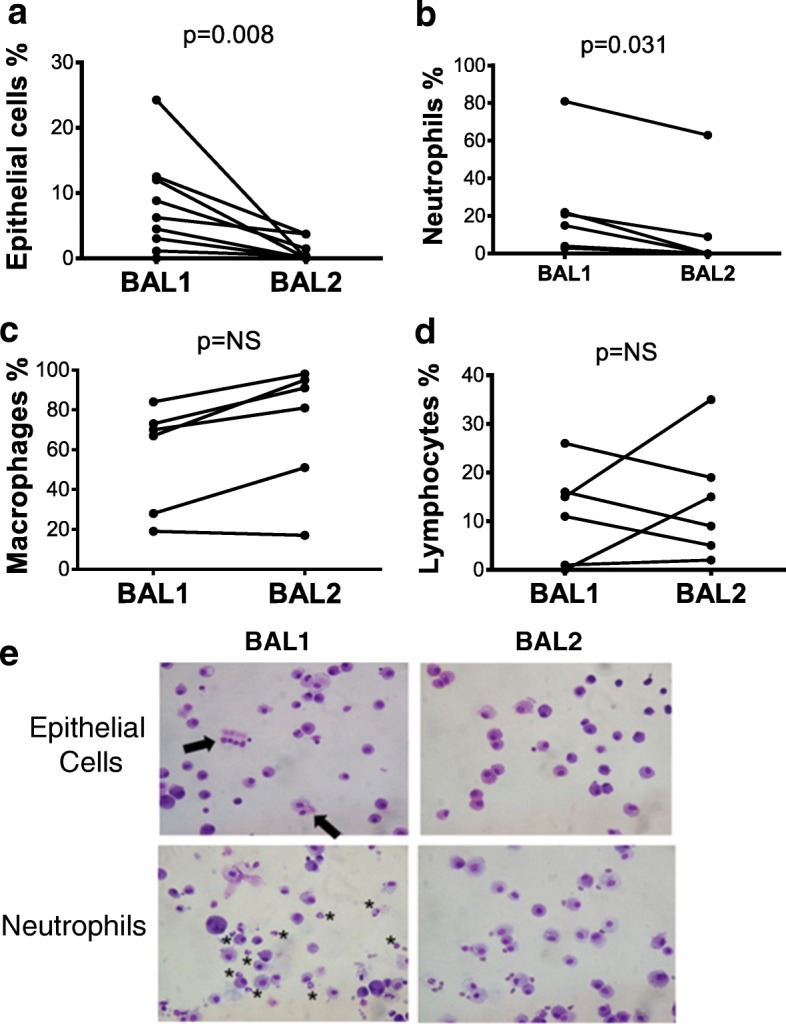
Differential Cell Counts in sequential bronchoalveolar lavages performed in lung transplant recipients. Two sequential bronchoalveolar lavages (BALs) were performed in 6 consecutive lung transplant recipients and cell subtypes were manually counted. Differential cell counts demonstrate that BAL2 has a lower proportion of epithelial cells (*p* < 0.05) (**a**), a lower proportion of neutrophils (*p* < 0.05) (**b**), a trend towards greater proportion of macrophages (*p* = 0.06) (**c**), and a similar proportion of lymphocytes (*p* = 0.78) (**d**). A non-parametric Wilcoxon matched-pairs signed rank test was used for comparison. Representative images (X200 magnification) of two sequential bronchoalveolar lavage (BAL1 & BAL2) cytospins stained with hematoxylin and eosin from lung transplant recipients showing abundance of epithelial cells (arrows) and neutrophils (stars) in BAL1 compared with BAL2 (**e**)

BAL1 contained a higher proportion of bronchial ciliated epithelial cells (6.25% (2.08, 12.25) vs. 0% (0, 2.60), for BAL1 and BAL2 respectively, *p* < 0.05) (Fig. 4a), more neutrophils (18% (3.75, 36.75) vs. 0 (0, 22.5), for BAL1 and BAL2 respectively, *p* < 0.05) (Fig. 4b), and a trend towards a lower percentage of macrophages (68.5% (25.75, 75.75) vs. 86% (42.5, 95.75), for BAL1 and BAL2 respectively, *p* = 0.06) (Fig. 4c). The proportion of lymphocytes in BAL1 and BAL2 was similar (13% (0.75, 18.5) vs. 12% (4.25, 23), for BAL1 and BAL2 respectively, *p* = 0.78) (Fig. 4d). Eosinophils were detected in only one BAL2 sample.

### BAL1 demonstrates increased CCSP levels whereas levels of SP-D are higher in BAL2

**Fig. 5:**
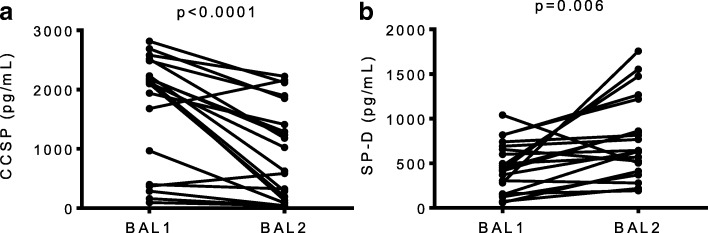
CCSP & SP-D levels in sequential bronchoalveolar lavages performed in lung transplant recipients. Two sequential bronchoalveolar lavages (BALs) were performed in 20 consecutive lung transplant recipients and levels of CCSP and SP-D were measured by enzyme-linked immunosorbent assay. BAL2 was found to contain a lower concentration of CCSP (*P* < 0.001) (**a**) and a higher concentration of SP-D (*P* < 0.05) (**b**) compared to BAL1. A non-parametric Wilcoxon matched-pairs signed rank test was used for comparison

We quantified CCSP, secreted by club cells of the airway epithelium, and SP-D, secreted primarily by type 2 alveolar cells, in a random set of paired BAL fractions. As shown in Fig. 5, the concentration of CCSP was significantly higher in BAL1 compared with BAL2 (2129 pg/ml (540.9, 2422) vs. 824.7 pg/ml (147, 1742), for BAL1 and BAL2 respectively, *p* = 0.0001) (Fig. 5a). The reverse was observed with SP-D, which was found to be considerably lower in BAL1 compared with BAL2 (437.8 pg/ml (188.8, 682.8) vs. 639.2 pg/ml (434.1, 1131), for BAL1 and BAL2 respectively, *p* < 0.05) (Fig. 5b and Additional file 1: Table S3).

### Differential levels of inflammatory markers were shown in the two BAL fractions

**Fig. 6:**
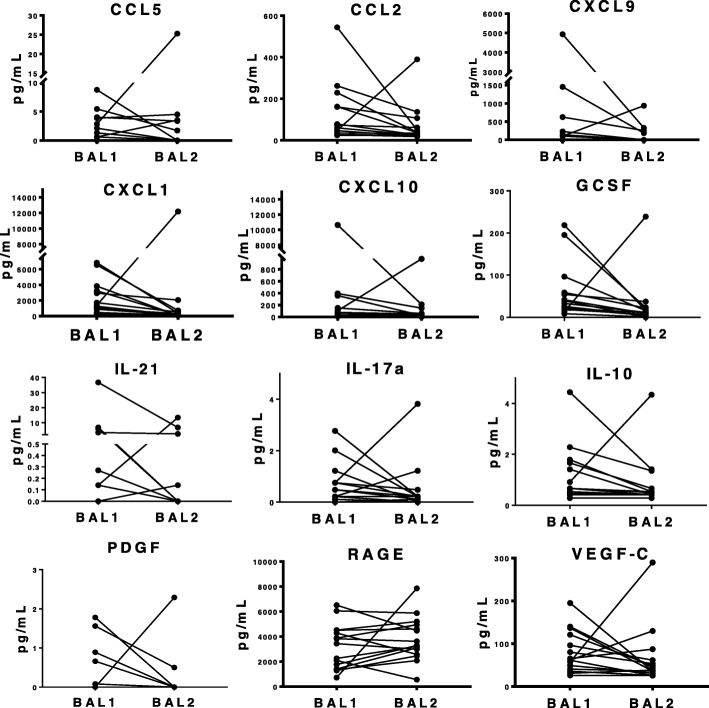
Levels of Inflammatory Markers in sequential bronchoalveolar lavages performed in lung transplant recipients. Two sequential bronchoalveolar lavages (BALs) were performed in 15 consecutive lung transplant recipients and levels of CXCL10, IL-10, CCL2, CCL5, VEGF-C, RAGE, CXCL9, CXCL1, IL-17A, IL-21, PDGF and GCSF were measured using a custom multiplex bead kit. While none of the comparisons were significant, there was a trend towards lower inflammatory markers in nearly all BAL2 samples compared to BAL1. A non-parametric Wilcoxon matched-pairs signed rank test was used for comparison

In a separate random set of paired BAL fractions, inflammatory markers CXCL10, IL-10, CCL2, CCL5, VEGF-C, RAGE, CXCL9, CXCL1, IL-17A, IL-21, PDGF, and GCSF were shown to be overall lower in BAL2 with different proteins following distinct patterns (Additional file 1: Table S3 and Fig. 6). Furthermore, the level of variability between samples appeared considerably lower in BAL2. As is frequently seen with BAL cytokine levels, analyte values were below detection in a subset of samples: concentrations of CCL5, CXCL9, IL-17A, IL-21 and PDGF were undetectable in greater than 50% of the samples and interpretation of this data is therefore limited. Nevertheless, all analytes were detectable in at least some samples. The percentage of undetectable analytes did not differ between BAL1 and BAL2. Almost all markers were lower in BAL2 compared to BAL1 for all paired samples, except for one, which consistently followed the opposite pattern. Although we cannot prove this, the outlier BAL pair may have resulted from an inadvertent switch of the two BAL fractions between sample collection and analysis. Given this rationale, while we included all BAL pairs in our primary analysis, we also compared BAL1 to BAL2 after exclusion of the outlier: when excluding the outlier, levels of VEGF-C, CXCL9, IL-17A, and IL-21 were statistically lower in BAL2, compared to BAL1.

### Correlation between BAL1 and BAL2

We assessed the correlation between BAL1 and BAL2 for all parameters. For example, there was a statistically significant correlation between BAL1 and BAL2 volumes (*r* = 0.58, *p* < 0.0001), meaning that if a patient had a high volume return in BAL1, they were likely to have a high return in BAL2 as well. Similarly, there was a statistically significant correlation between BAL1 and BAL2 total cell concentrations, cell viability, macrophage concentration, CCSP, SP-D and most of the other measured proteins. For each analysis, *r* and *p* values are reported in Additional file 1: Tables S2 and S3.

## Discussion

In this study, we assessed the cellular and soluble protein composition of sequential BAL fractions in lung transplant recipients. We showed that there are systematic differences between BAL1 and BAL2, with BAL1 preferentially reflecting the airway compartment and BAL2 composition being consistent with the distal bronchoalveolar space. To the best of our knowledge, this is the first study delineating unique compositions of sequential BALs in lung transplantation.

BAL cells and proteins have long been studied in the context of pulmonary diseases. In lung transplantation, in particular, BAL parameters have been shown to correlate with allograft dysfunction [7]. However, concerns exist regarding the lack of standardization of BAL techniques, which hinders the potential clinical utility of measured BAL components and limits comparison and potential for collaboration between centers. Our data indicate that the sequence of BAL fractions and their subsequent pooling may significantly alter results.

The results of this study demonstrate reproducible differences between sequential fractions of BAL. The volume recovery was consistently higher for BAL2 independent of other factors such as CLAD status, known to influence volume recovery [27]. Further, BAL1 is enriched with dead cells, airway epithelial cells, and neutrophils. This suggests that the first fraction may be a good representation of the airway compartment. BAL2 appears to better represent the more distal part of the lung, including the alveoli: it contains less airway epithelial cells and more macrophages (although the macrophage difference is not statistically significant). The concept of sequential BALs representing distinct compartments of the lung has been brought forth in the past in a study by Kelly et al. who was able to directly visualize the increasingly distal anatomical distribution of three sequential 60 ml aliquots of saline using a radio-opaque dye in a subtraction imaging technique [28]. This is further supported by our finding of higher CCSP levels and lower SP-D levels in BAL1 compared to BAL2. CCSP is a major secretory product of club cells found in the airway epithelium, making it a useful marker of the airway compartment. Conversely, SP-D is primarily synthesized and secreted by alveolar type II cells [29] that line the alveolar spaces potentially explaining the higher level found in BAL2. Unlike CCSP and SP-D, CXCL10, IL-10, CCL2, CCL5, VEGF-C, RAGE, CXCL9, CXCL1, IL-17A, IL-21, PDGF, and GCSF, that have previously been shown to correlate with clinical outcomes in lung transplant recipients, are not known to be secreted preferentially in the proximal airways or distal alveolar space. The higher levels of these proteins in BAL1 may reflect a more significant contribution of the distal airways to their production or merely a dilution effect in BAL2. Of note, the protein analyses were performed on a small and randomly selected subset of samples and are not representative of any specific post lung-transplant conditions.

Our observations regarding the cellular and soluble components of sequential BALs are consistent with several studies carried out in non-transplant populations in the 1980’s [9, 30–35]. A small volume lavage (less than 20 ml) in the mainstem bronchus or a segmental bronchus recovered more epithelial cells and neutrophils, while a larger lavage volume of 20-100 ml in a segmental bronchus recovered more alveolar macrophages [31], which is in line with our data. Unlike the higher level of alveolar lymphocytes that was described by Lam et al. in BAL from a segmental bronchus [31], we did not detect any particular trend in the proportion of lymphocytes between BAL1 and BAL2. As in some of the prior studies in non-transplant patients [30, 32], our results show a decrease in protein concentration in successive BAL fractions. A possible explanation could be the influence of dilution and volume returns [2], as volumes recovered from BAL2 were consistently higher than BAL1. However, a preferential sampling of different lung compartments also likely plays a role as SP-D levels were higher in BAL2. Our results add to the literature by validating and expanding findings from earlier smaller studies in a large cohort of lung transplant patients undergoing mostly surveillance bronchoscopies. Furthermore, these findings are directly applicable to the care of both lung transplant recipients as well as to patients with other pulmonary conditions. The significant differences in cellular composition and variations in soluble proteins between BAL1 and BAL2 lead to the important conclusion that sequential BALs should not be used interchangeably. Inconsistent data collection (i.e., one study using BAL1 for analysis and the other using BAL2 or pooled sequential BALs) would make comparisons between studies unreliable. Standardization of collection, processing and reporting methods is essential for clear communication among medical professionals and researchers.

Several limitations warrant review in discussion of this work. The results described in this study should be interpreted in the context of a center-specific protocolized BAL collection method as described herein. We were not able to assess the impact of other BAL practices such as instillation of a higher number of fractions or different volumes, pooled BAL1 and 2 as opposed to BAL1 and BAL2, the use of different BAL locations within the same lung or between lungs, or variability in sample handling, on the difference in composition between BAL1 and BAL2. In addition, according to our protocol a large airway bronchial wash (LABW) of the mainstem bronchus is performed prior to BAL. As shown by others, the LABW composition differs from BAL in cell and protein composition [31]. It is possible that that performing a LABW prior to retrieving BAL1 may influence the composition of BAL1 by introducing more dead cells and higher protein levels. A separate study to compare BAL composition in the presence or absence of LABW is necessary to address this question. Furthermore, since microbiology analysis is performed only on BAL1 at our center, we were not able to compare the pathogen recovery between BAL1 and BAL2, which could be valuable information. An important issue in BAL protein analysis is the normalization of proteins diluted by saline relative to the return. Different methods have been proposed such as normalization to albumin or urea in BAL versus serum; published data suggests that albumin is not a reliable marker while urea may be useful for that purpose [36, 37]. Since there is no consensus about an optimal normalization strategy and given that the majority of publications on BAL proteins in lung transplantation do not use a normalization approach, we reported simple unadjusted concentrations of the protein elements and did not employ any correction methods to account for changes in dilutions between BAL1 and BAL2. In light of the critical importance that dilution may have on BAL composition, a standardized dilution should be used consistently as a part of a BAL collection protocol. We acknowledge that biochemical analyses in this study were done on small sample size. In spite of the low patient numbers in some analyses, we were able to detect changes between BAL1 and BAL2 which are consistent with previous reports described in biochemical analyses of sequential bronchial lavages from healthy volunteers [30, 32]. Another point deserving consideration is that this study population only included lung transplant patients: The study design did not include a comparison population (patients with other pulmonary conditions or alternatively healthy volunteers), which hinders generalizations of our observations to other patient populations. Additionally, we acknowledge that specific post-transplant complications, such as CLAD status, presence of acute rejection or infection, degree of immunosuppression, and others, may alter cell and protein composition as well as BAL fluid recovery. Our study was not focused on assessing the relationship between these factors and the BAL analytes. Given our paired study design with subjects acting as their own controls, comparing BAL1 to BAL2 in each individual subject, we were able to minimize the potential confounding effects of clinical variables on the primary analysis.

## Conclusion

BAL cell composition and protein concentrations in lung transplant recipients are influenced by regional sampling and dilution factors that characterize different sequential BAL fractions. The increasing interest in BAL as a research tool in pulmonary translational research merits standardization of its collection, processing, bio-banking, and thorough description in manuscripts. Consensus guidelines for the collection and processing of BAL are needed for greater uniformity in future study protocol development.

## Additional file

Additional file 1: **Table S1.** Variation in bronchoalveolar lavage collection between different institutions. **Table S2.** Cell concentrations and differential counts in sequential bronchoalveolar lavage samples in lung transplant recipients. **Table S3.** Concentrations of selected proteins in sequential bronchoalveolar lavage samples in lung transplant recipients. (DOCX 51 kb)

## Abbreviations

BAL: Bronchoalveolar lavage
CCSP: Club cell secretory protein
CLAD: Chronic lung allograft dysfunction
ELISA: Enzyme-linked immunosorbent assay
LABW: Large airway bronchial wash
SP-D: Surfactant protein D
