# Supplementary material for: Sequential broncho-alveolar lavages reflect distinct pulmonary compartments: clinical and research implications in lung transplantation
Source: Respir Res. 2018 May 25;19:102. doi: 10.1186/s12931-018-0786-z (PMC5970521; doi:10.1186/s12931-018-0786-z)
Supplement: Supplementary file 1 — Table S1. Variation in bronchoalveolar lavage collection between different institutions. Table S2. Cell concentrations and differential counts in sequential bronchoalveolar lavage samples in lung transplant recipients. Table S3. Concentrations of selected proteins in sequential bronchoalveolar lavage samples in lung transplant recipients. (DOCX 51 kb) [file 12931_2018_786_MOESM1_ESM.docx]

Table S1. **Variation in bronchoalveolar lavage collection between different institutions**

| Anonymized Institution No. | Volume Instilled (ml) | Number of Sequential BALs | Total Volume instilled (ml) | Pooling of BAL samples |
| --- | --- | --- | --- | --- |
| 1 | 50 | 2 | 100 | No |
| 2 | 60 & 20 | 2 | 80 | No |
| 3 | 60 | 3 | 180 | Yes |
| 4 | 20 | 3 | 60 | Yes |
| 5 | 25 | 3-6 | 100-150 | Yes |
| 6 | 20 | 3 | 60 | Yes |
| 7 | 50 | 2 | 100 | Yes |
| 8 | 60 | 3 | 180 | Yes |
| 9 | 20 | 3 | 60 | No |
| 10 | 50 | 3 | 150 | Unknown |
| 11 | 50 | 3 | 150 | Unknown |
| 12 | 20 | 5 | 100 | Yes |
| 13 | 20X2 & 50X3 |  | 190 | Unknown |
| 14 | 50 | 2 | 100 | Unknown |
| 15 | 20 | 5 | 100 | Yes |
| 16 | 30 | 4 | 120 | Unknown |
| 17 | 50 | 4 | 200 | Unknown |
| 18 | 50 | 2 | 100 | Unknown |
| 19 | 30 | 3 | 90 | Sometimes |
| 20 | 60 | 1 | 60 | Yes |
| 21 | 60 | 2-3 | 120-180 | Sometimes |
| 22 | 50 | 3 | 150 | Sometimes |
| 23 | 100-200 | 1 | 100-200 | Sometimes |
| 24 | 100 | 1 | 100 | Sometimes |

Table S2. **Cell concentrations and differential counts in sequential bronchoalveolar lavage samples in lung transplant recipients.**

|  | **Paired comparison of**  **BAL1 and BAL2** | | | **Correlation between BAL1 and BAL2** | |
| --- | --- | --- | --- | --- | --- |
| **Total cell concentration *n*=122** | **BAL1** | **BAL2** | ***p*-value** | ***r*** | ***p-value*** |
| Cell concentration (×10^6^/ml) | 0.32 [0.17, 0.67] | 0.27 [0.16, 0.54] | NS | 0.64 | <0.0001 |
| **Cell viability** (%) ***n*=9** | **BAL1** | **BAL2** | ***p*-value** | ***r*** | ***p-value*** |
| Cell viability | 88.89[85.92, 95.18] | 96.43 [95.83, 98.92] | 0.008 | 0.31 | 0.43 |
| **Epithelial Cell Count (%) *n*=9** | **BAL1** | **BAL2** | ***p*-value** | ***r*** | ***p-value*** |
| Epithelial cells | 6.25 [2.08, 12.25] | 0 [0, 2.6] | 0.008 | 0.21 | 0.29 |
| **Differential Cell Count (%) *n*=6** | **BAL1** | **BAL2** | ***p*-value** | ***r*** | ***p-value*** |
| Macrophages | 68.5 [25.75, 75.75] | 86 [42.5, 95.75] | 0.06 | 0.82 | 0.03 |
| Lymphocytes | 13 [0.75, 18.5] | 12 [4.25, 23] | NS | 0.43 | 0.21 |
| Neutrophils | 18 [3.75, 36.75] | 0 [0, 22.5] | 0.03 | 0.68 | 0.1 |
| Eosinophils | 0 [0,0] | 0 [0, 0.25] | NS | NA | NA |

Values are presented as median [IQR]. NS = non-significant. NA = not applicable.

Table S3. **Concentrations of selected proteins in sequential bronchoalveolar lavage samples in lung transplant recipients.**

|  | **Sample size** | **Concentration (pg/ml)** | | | **Correlation between BAL1 and BAL2** | |
| --- | --- | --- | --- | --- | --- | --- |
|  |  | **BAL1** | **BAL2** | ***p*-value** | ***r*** | ***p-value*** |
| CCSP | 20 | 2129 [540.9, 2422] | 824.7 [147, 1742] | 0.0001 | 0.71 | 0.0002 |
| SP-D | 20 | 437.8 [188.8, 682.8] | 639.2 [434.1, 1131] | 0.002 | 0.41 | 0.04 |
| CXCL10  IL-10  CCL2  CCL5  VEGF-C  RAGE  CXCL9  CXCL1  GCSF  IL-17A  IL-21  PDGF | 15  15  15  15  15  15  15  15  15  15  15  15 | 61.16 [9.47, 150.2]  0.66 [0.47, 1.66]  59.6 [28.44, 162.1]  0.61 [0, 3.86]  61.52 [40.5, 121.1]  3441 [1408, 4514]  0 [0, 229.1]  1269 [884.6, 3254]  33.17 [18.58, 58.85]  0.48 [0.11, 0.75]  0.14 [0, 3.46]  0 [0, 0.66] | 20.93 [5.17, 58.02]  0.41 [0.41, 0.66]  34.33 [22.82, 61.89]  0 [0, 3.36]  38.29 [31.12, 61.52]  3287 [2574, 4949]  0 [0, 185.5]  144.5 [103.4, 518.7]  11.82 [4.79, 22.25]  0.22 [0, 0.22]  0 [0, 0.14]  0 [0,0] | 0.007  0.03  0.007  NS  NS*  NS  NS*  0.008  0.008  NS*  NS*  NS | 0.88  0.84  0.69  0.55  0.33  0.42  0.7  0.48  0.39  0.47  0.21  0.15 | <0.0001  <0.0001  0.003  0.02  NS  NS  0.005  0.04  NS*  0.04  NS  NS |

* The asterisks indicate p-values that become significant (<0.05) after excluding the outlier, as explained in the text.
